# Digital blindness: the hidden cost of excessive screen time

**DOI:** 10.17179/excli2024-7041

**Published:** 2024-04-30

**Authors:** Nikhil Aggarwal, Noor Us Saba

**Affiliations:** 1 Department of Anatomy, Maulana Azad Medical College, New Delhi - 110002, India; 2 King George's Medical University UP, Lucknow, India

## ⁯⁯

Digital blindness is a term used to describe the visual discomfort and strain caused by excessive exposure to digital screens. A blue light, emitted by digital screens, has shorter wavelengths and higher energy levels than other colors in the visible light spectrum, making it the main culprit for this problem. Poor ergonomics, improper light conditions in the surroundings, and excessive glare of digital screens can contribute to the issue more cogently. To protect the eyes in the digital age, a 20-20-20 rule has been proposed with marked adjustments of screen settings in the workplace and optimizing workstation ergonomics. These efforts can assist in reducing strain on the eyes as well as reduction of pain and rigidity in the neck and shoulder regions. Regular blinking of eyes, frequent breaks from screen time, improved surrounding lights and periodic eye check-ups are all useful strategies to reduce the risk of developing digital eye strain. It is always essential to give priority to health, particularly needed for vision, to create a balance between technology and the well-being of the individuals.

In today's world, with advanced technologies, digital devices have become an inseparable part of humans' day-to-day life. Everyone is constantly exposed to screens in the form of smartphones, computers, tablets and television. Inevitable exposure of digital rays adversely affects almost every part of the human body. Effects can be neck and shoulder pain, backache, unreasonable headaches, eye strain, as well as a sedentary lifestyle contributing to weight gain. Ophthalmological problems, developed due to eye strain, which is caused by excessive exposure to digital screens, are often referred as “digital blindness.” Digital blindness, while not a medical term, is a metaphorical expression used to describe the visual discomfort and strain caused by excessive exposure to digital devices. A potential negative impact on the eye due to prolonged use of screens at the workplace by many professionals has always raised a huge concern globally. This article delves into the concept of digital blindness, exploring the causes, and highlighting the methods to protect vision and health of the eyes in the digital age.

Several factors contribute to the development of digital blindness, of which the exposure of blue light emitted by digital screens is a major concern. This blue light has a higher energy level due to its shorter wavelength, compared to other colors in the visible spectrum of light. Disrupted sleep patterns, leading to daytime fatigue due to sleep disturbances, are another effect seen from blue light which is emitted by the device (Tosini et al., 2016[4]). A marked reduction in eye blinking during work on the screen, might also bring irritation in the eyes because of dryness (Sheppard and Wolffsohn, 2018[3]). Poor ergonomics including improper surrounding lights and excessive glare from screens further contribute to visual fatigue, strain and discomfort in the eyes.

### Methods to protect eyes in digital age

#### Follow 20-20-20 Rule

During screen time, every 20 minutes, giving a break to the eyes to see a real object for 20 seconds which should be there at about 20 feet away from the eye. This practice favors relieving eye strain and provides an opportunity to relax and refocus after every 20 minutes of digital exposure.

#### Adjust Screen Settings

Lowering the brightness of the screen, selecting larger and adequate font, and adjusting the device's color contrast can help to minimize overall eye strain. Blockage or filtration of harmful blue light using specialized glasses is another way to protect the eyes at the workplace. 

#### Optimize Workstation Ergonomics

The significance of ergonomics in decreasing musculoskeletal pain and eye strain has already been acknowledged (Murata and Shibuya, 2016[2]). So, a proper sitting posture to maintain the level of the screen just at the level of one's eyes and ensuring a comfortable distance from the screen are prerequisites for a significant decline in eye-related symptoms along with straining in the neck, shoulder, and back muscles. 

#### Blink Frequently

Eye blinking at a normal frequency helps to keep the eyes moist and reduces dryness. A unique recommendation is to sustain a regular blinking pattern, which helps maintain the stability of tear film between eyeball and eyelids. A moist eye, aided by the tear film, discourages dryness and subsequent irritation in the eyes (Wang et al., 2018[5]).

#### Take Regular Breaks

Implementing a constructive routine that includes scheduling short breaks from screen work throughout the day provides ample rest for the eyes. Engaging more in activities that do not involve digital screens can also help reduce digital eye strain (Kaur et al., 2022[1]).

#### Improve Lighting Conditions

Minimizing glare by adjusting ambient light and avoiding excessive brightness from screens and surrounding light sources can significantly reduce eye strain and discomfort.

#### Get Regular Eye Check-Ups

Regular ophthalmological examinations can detect impending eye related issues before it affects the vision of a person. Visiting an eye care professional, to monitor and address any underlying issues or concerns, is essential in detecting and managing potential eye problems associated with the use of technical digital device. 

In the age of advancing technologies, excessive use of digital screens can lead to discomfort, fatigue, and other eye-related symptoms. These symptoms can be managed by implementing and sustaining healthy routine at workplace in context to the use of screen. The phenomenon of digital blindness affecting a significant portion of the population due to prolonged hours of screen use necessitates control measures to mitigate the harmful effects of technology. Priority should be given to maintain healthy eyes which is crucial to strike a balance between benefits of technology and well-being of the individuals.
